# Interactive effects of drought and heat stresses on morpho-physiological attributes, yield, nutrient uptake and oxidative status in maize hybrids

**DOI:** 10.1038/s41598-019-40362-7

**Published:** 2019-03-07

**Authors:** Hafiz Athar Hussain, Shengnan Men, Saddam Hussain, Yinglong Chen, Shafaqat Ali, Sai Zhang, Kangping Zhang, Yan Li, Qiwen Xu, Changqing Liao, Longchang Wang

**Affiliations:** 1 Key Laboratory of Eco-environments in Three Gorges Reservoir Region/Engineering Research Center of South Upland Agriculture, Ministry of Education/College of Agronomy and Biotechnology, Southwest University, Chongqing, 400715 China; 2 Institute of Environment and Sustainable Development in Agriculture, Chinese Academy of Agricultural Sciences/Key Laboratory of Agro-Environment, Ministry of Agriculture, Beijing 100081, China; 3 Department of Agronomy, University of Agriculture, Faisalabad, 38040 Punjab Pakistan; 4 Institute of Soil and Water Conservation, Chinese Academy of Sciences, and Northwest A&F University, Yangling, 712100 China; 5 Institute of Agriculture, and School of Agriculture and Environment, The University of Western Australia, Perth, WA 6009 Australia; 6 Department of Environmental Sciences and Engineering, Government College University, Faisalabad, 38000 Pakistan

## Abstract

Maize is a sensitive crop to drought and heat stresses, particularly at the reproductive stages of development. The present study investigated the individual and interactive effects of drought (50% field capacity) and heat (38 °C/30 °C) stresses on morpho-physiological growth, yield, nutrient uptake and oxidative metabolism in two maize hybrids i.e., ‘Xida 889’ and ‘Xida 319’. The stress treatments were applied at tasseling stage for 15 days. Drought, heat and drought + heat stress caused oxidative stress by the over-production of ROS (O^2−^, H_2_O_2_, OH^−^) and enhanced malondialdehyde contents, which led to reduced photosynthetic components, nutrients uptake and yield attributes. The concurrent occurrence of drought and heat was more severe for maize growth than the single stress. However, both stresses induced the metabolites accumulation and enzymatic and non-enzymatic antioxidants to prevent the oxidative damage. The performance of Xida 899 was more prominent than the Xida 319. The greater tolerance of Xida 889 to heat and drought stresses was attributed to strong antioxidant defense system, higher osmolyte accumulation, and maintenance of photosynthetic pigments and nutrient balance compared with Xida 319.

## Introduction

Under natural environments, crops are often subjected to different abiotic stresses simultaneously during their life cycle which adversely affect the growth and productivity of field crops^1,2^. Among abiotic stresses, extreme temperature and water deficit conditions are two of the most frequent environmental threats to crop growth and productivity, and ultimately the food security under changing climate^3–8^. These stresses negatively affect the yield of major staple food crops^5,6,9^, that account for 60% of global food energy supply^10^. Previous studies showed that the combined drought and heat stresses caused a disproportionate damage on plant growth and productivity compared with each of individual stress^11–15^. Temperature above 35 °C affects the vegetative and reproductive growth of maize, from germination to grain filling^16^. However, the reproductive stages of the crop plants are more sensitive to combined drought and heat stresses than the vegetative stages, although each stress affected reproductive traits differently^12,15^. These stresses noticeably reduced the photosynthetic activity, altered oxidative metabolism, caused membrane instability^7,17^, affected stomatal conductance, and decreased the leaf area and water-use efficiency in maize and other cereal crops^5,6,18^. Nonetheless, the effects of these stress factors might be different depending on the plant species^2^.

Decreased and uneven precipitation can lead to more severe drought events, and high air temperature coupled with drought results in higher plant tissue temperature^19^. Wahid *et al*.^20^ stated that the heat stress can be accompanied by drought because of rapid water loss from plant and soil surface under high temperature. While, Pie *et al*.^21^ reported that heat stress may occur in plants due to insufficient water supply to meet the evaporative demand. Both drought and heat stresses are well known to decrease the the nutrient uptake and photosynthetic efficiency of plants^22^. In wheat crop, heat stress during early as well as late season disturbed the growth period for various development stages (e.g. tillering, jointing, booting, anthesis, and grain filling) and reduced the organ size (tiller, leaf, and spikes)^23^. Recently, Zandalinas *et al*.^2^ and Lamaoui *et al*.^22^ concluded that drought and heat stresses generally trigger similar physiological responses in plants, however, plants were severely damaged by the combined action of both stresses than by the individual stress factor, indicating that conserved defense mechanisms exist among different plant species to deal with a combination of heat and drought stresses. In order to adapt stress conditions, plants possess several mechanisms including accumulation of compatible solutes (e.g. sugars, proline) and protective proteins (e.g. heat shock proteins), and activation of enzymatic (total superoxide dismutase; T-SOD, catalase; CAT, peroxidase; POD, ascorbate peroxidase; APX) and non-enzymatic (e.g. reduced glutathione GSH) antioxidant systems^1,24,25^. However, it is worthy mentioned that the stress sequence imposition of heat and drought do not alter the global response to combined stresses^2^.

Maize (*Zea mays* L.) is one of the most important and widely grown crops in the world^26^. However, in many parts of the world, it is mainly cultivated in semi-arid environments and thus often faces high temperature, water scarcity, and a combination of these factors in field conditions^27,28^. Maize is originated from the tropics but is still sensitive to heat and drought stresses, particularly after 8^th^ leaf stage^29^. In China, 60% of crops in maize growing regions are often subjected to heat and drought spells, which may result in 30% yield losses per year^28^. Owing to changing climatic patterns worldwide, it is projected that these stresses will become major threats to maize yields and will decrease the world maize production by 15–20% each year^30,31^.

Despite the frequent existence of combined heat and drought stress episodes under field conditions, most of the previous studies have focused on independent drought or heat stress responses in plants^15^. Therefore, little is known on the morpho-physiological and biochemical responses of maize to combined drought and heat stresses. The present study was aimed at elucidating the mechanism of combined drought and heat stress tolerance in maize hybrids by exploring the physiological and biochemical responses of plants along with grain yield. The specific objectives of the study were (1) to investigate the individual and concurrent effects of heat and drought stresses on morpho-physiological growth, grain yield, osmolyte accumulation, nutrient uptake and oxidative status in maize, (2) to examine the basis of maize tolerance against drought and heat stresses, and (3) to assess the performance of two different maize hybrids under drought and/or heat stresses.

## Results

### Growth and yield

Drought and heat stress conditions alone or in combination hampered the morphological growth, grain yield and yield related attributes of both maize cultivars. Compared with control, combined drought + heat stress significantly reduced the plant height, shoot fresh weight, shoot dry weight, stem diameter, leaf area, kernels/ear, 100-kernel weight, grain yield/plant and harvest index (HI) of two maize hybrids (Tables 1 and 2). However, ears/plant and kernel rows/ear in both maize hybrids remained unaffected by all stress treatments (Table 2). At individual level, the effects of drought stress were more severe for plant height, stem diameter, shoot fresh weight, shoot dry weight, leaf area, 100-kernel weight, and grain yield/plant of both hybrids compared with those of heat stress. Under all stress treatments, the growth and yield performance of Xida 319 was lower than Xida 889 (Tables 1 and 2).

**Table 1:** Effect of drought and heat stresses on maize agronomic traits.

Maize cultivars	Treatments	Plant height (cm)	Stem diameter (mm)	Leaf area (cm^2^)	Shoot fresh weight/plant (g)	Shoot dry weight/plant (g)
Xida 319	Ck	181.67 ± 3.28	22.84 ± 0.70	455.87 ± 20.13	216.20 ± 7.63	51.43 ± 1.82
	H	165.67 ± 2.96*	22.59 ± 0.69	438.54 ± 20.88	209.10 ± 2.29	49.50 ± 3.50
	D	163.33 ± 2.85*	20.48 ± 0.92	435.07 ± 22.52	190.43 ± 7.47*	42.87 ± 1.95*
	H + D	154.87 ± 6.26**	19.06 ± 0.60**	386.19 ± 9.13*	173.30 ± 6.36**	41.90 ± 1.63*
	Means	166.38	21.24	428.92	197.26	46.43
Xida 889	Ck	185.67 ± 3.76	23.34 ± 0.22	542.73 ± 6.80	233.83 ± 10.08	53.67 ± 1.86
	H	173.67 ± 2.19*	22.63 ± 0.11	517.11 ± 15.21	214.93 ± 8.03	51.87 ± 0.85
	D	170.50 ± 4.80*	20.92 ± 0.40*	494.68 ± 4.22*	191.33 ± 10.84*	48.13 ± 1.82*
	H + D	158.33 ± 2.85**	20.25 ± 1.08**	465.37 ± 15.31**	178.90 ± 8.59**	46.23 ± 1.13*
	Means	172.04	21.79	504.98	204.75	49.98

Values are means of three replicates ±SE. For Duncan’s results, means with asterisks are considered as significant different compared with control. *P ≤ 0.05; **P ≤ 0.01.

Ck, control; H, heat stress; D, drought stress and H + D, heat + drought stress.

**Table 2:** Effect of drought and heat stresses on maize yield and related characteristics.

Maize cultivars	Treatments	Ears/plant	Kernel rows/ear	Kernels/ear	100-kernel weight(g)	Grain yield/plant(g)	HI
Xida 319	Ck	2.02 ± 0.09	14.32 ± 0.36	281.33 ± 9.26	22.97 ± 0.37	107.33 ± 4.98	0.30 ± 0.01
	H	1.93 ± 0.05	13.89 ± 0.23	248.70 ± 6.67*	22.20 ± 0.44	89.50 ± 5.07*	0.25 ± 0.01*
	D	1.87 ± 0.11	13.95 ± 0.54	237.67 ± 11.35**	21.97 ± 0.90	85.20 ± 7.16*	0.24 ± 0.01*
	H + D	1.82 ± 0.06	13.51 ± 0.51	202.67 ± 3.71**	18.67 ± 0.33**	72.70 ± 2.76**	0.20 ± 0.02**
	Means	1.91	13.92	242.59	21.45	88.69	0.25
Xida 889	Ck	2.11 ± 0.05	14.86 ± 0.55	308.00 ± 9.02	23.93 ± 0.62	117.07 ± 5.53	0.33 ± 0.03
	H	1.94 ± 0.06	14.05 ± 0.53	266.00 ± 4.16*	22.63 ± 1.16	100.03 ± 3.18*	0.28 ± 0.01
	D	1.94 ± 0.03	13.67 ± 0.64	261.70 ± 4.44*	21.50 ± 0.76	96.10 ± 4.80*	0.26 ± 0.02*
	H + D	1.96 ± 0.05	13.97 ± 0.09	221.43 ± 15.50**	20.02 ± 0.58*	90.97 ± 4.98**	0.23 ± 0.01*
	Means	1.99	14.14	264.28	22.02	101.04	0.27

Values are means of three replicates ±SE. For Duncan’s results, means with asterisks are considered as significant different compared with control. *P ≤ 0.05; **P ≤ 0.01.

Ck, control; H, heat stress; D, drought stress and H + D, heat + drought stress.

### Photosynthetic characteristics

**Figure 1:**
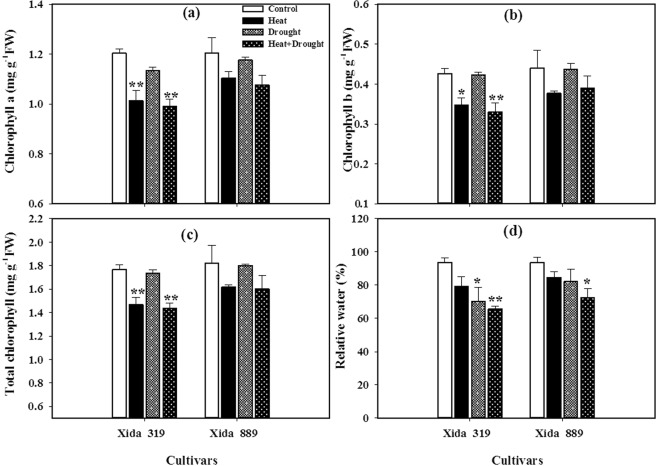
Influence of heat, drought, and heat + drought stresses on **(a)** chlorophyll a content, **(b)** chlorophyll b content, **(c)** total chlorophyll content and **(d)** relative water content in two maize hybrids. Capped bars above means represent ± SE of three replicates. Asterisks above columns means denote the significant differences compared with control treatment for a single maize hybrid. *P ≤ 0.05; **P ≤ 0.01.

The leaf chlorophyll concentration of maize hybrids decreased under stress conditions as compared with control. However, the negative effects of heat were more severe for chlorophyll contents than the individual effects of drought stress (Fig. 1). Compared with control, heat stress alone or in combination with drought significantly declined the chlorophyll a (Chl a), chlorophyll b (Chl b), and total chlorophyll content in Xida 319, which indicated its sensitivity under stress conditions (Fig. 1a–c).

Heat stress resulted in a slight decrease of relative water contents (RWC) in both maize hybrids, and these values were statistically similar with control. However, drought stress significantly reduced the RWC in Xida 319, which were further decreased under drought + heat stress. The reduction of RWC in Xida 319 was greater under drought + heat stress than that in Xida 889 (Fig. 1d).

**Figure 2:**
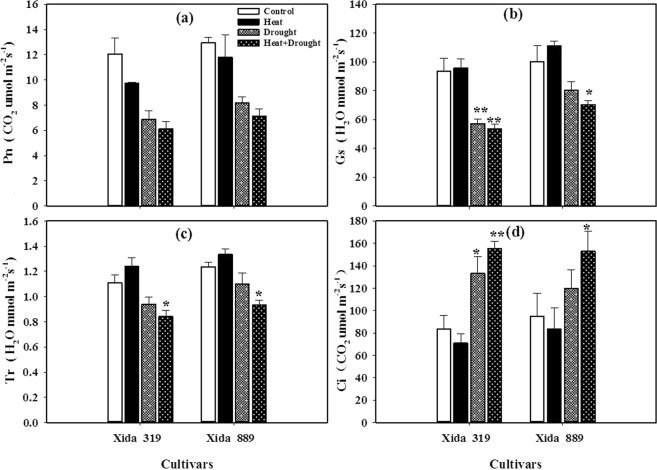
Influence of heat, drought, and heat + drought stresses on **(a)** photosynthetic rate (Pn), **(b)** stomatal conductance (Gs), **(c)** transpiration rate (Tr) and **(d)** intercellular CO_2_ (Ci) in two maize hybrids. Capped bars above means represent ± SE of three replicates. Asterisks above columns means denote the significant differences compared with control treatment for a single maize hybrid. *P ≤ 0.05; **P ≤ 0.01.

The photosynthetic rate (Pn), transpiration rate (Tr), and stomatal conductance (Gs) in both Xida 319 and Xida 889 hybrids were reduced by drought and drought + heat stresses. However, the reductions in these traits were more severe under combined drought + heat stresses than drought stress individually (Fig. 2a–c). As compared with control, the levels of Gs and Tr were slightly increased by heat stress in both hybrids, but these effects were statistically non-significant. The intercellular CO_2_ concentration (Ci) was increased under drought and significantly enhanced by drought + heat stresses, while it was decreased slightly under heat stress (Fig. 2d). The drought and heat stresses alone or in combination did not have the same effects on photosynthetic gas exchange parameters (Fig. 2). For instance, Tr was increased by heat stress, while drought stress decreased it, and Tr was further decreased when plants were subjected to combined drought + heat stresses. Overall, Xida 889 showed greater photosynthetic ability compared with Xida 319 (Fig. 2).

### Reactive oxygen species and lipid peroxidation

**Figure 3:**
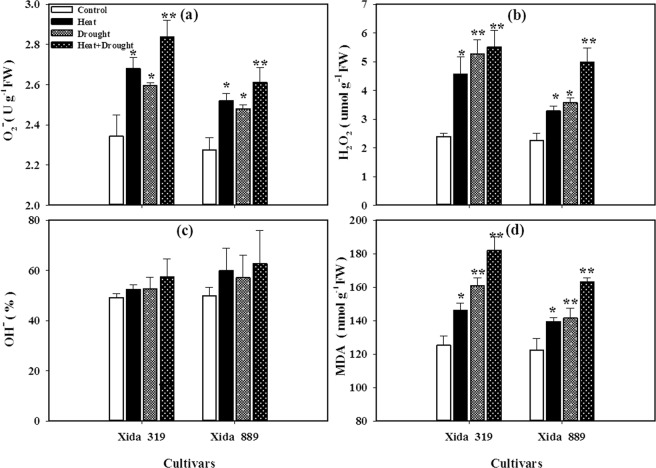
Influence of heat, drought, and heat + drought stresses on **(a)** superoxide anion (O_2_^−^), **(b)** hydrogen peroxide (H_2_O_2_), **(c)** hydroxyl radical (OH^**−**^) and **(d)** malonaldehyde (MDA) contents in two maize hybrids. Capped bars above means represent ± SE of three replicates. Asterisks above columns means denote the significant differences compared with control treatment for a single maize hybrid. *P ≤ 0.05; **P ≤ 0.01.

The reactive oxygen species (ROS) accumulation and the levels of membrane damage in two maize hybrids were increased under all stress conditions. However, the negative influence of drought + heat stress was more pronounced than those of individual stresses. The concentrations of hydroxyl free radical (OH^−^) in both hybrids under stress conditions were statistically similar with control. At individual stress level, the superoxide anion radical (O_2_^−^) contents were higher under heat stress, while hydrogen peroxide (H_2_O_2_) contents were higher under drought stress (Fig. 3). Compared with the control, drought, heat and drought + heat stress treatments significantly increased the levels of H_2_O_2_ and malondialdehyde (MDA) in both hybrids. Nevertheless, higher oxidative damage in Xida 319 indicated that this cultivar is less tolerant to heat and drought stresses than Xida 889 (Fig. 3).

### Activities/levels of antioxidants

**Figure 4:**
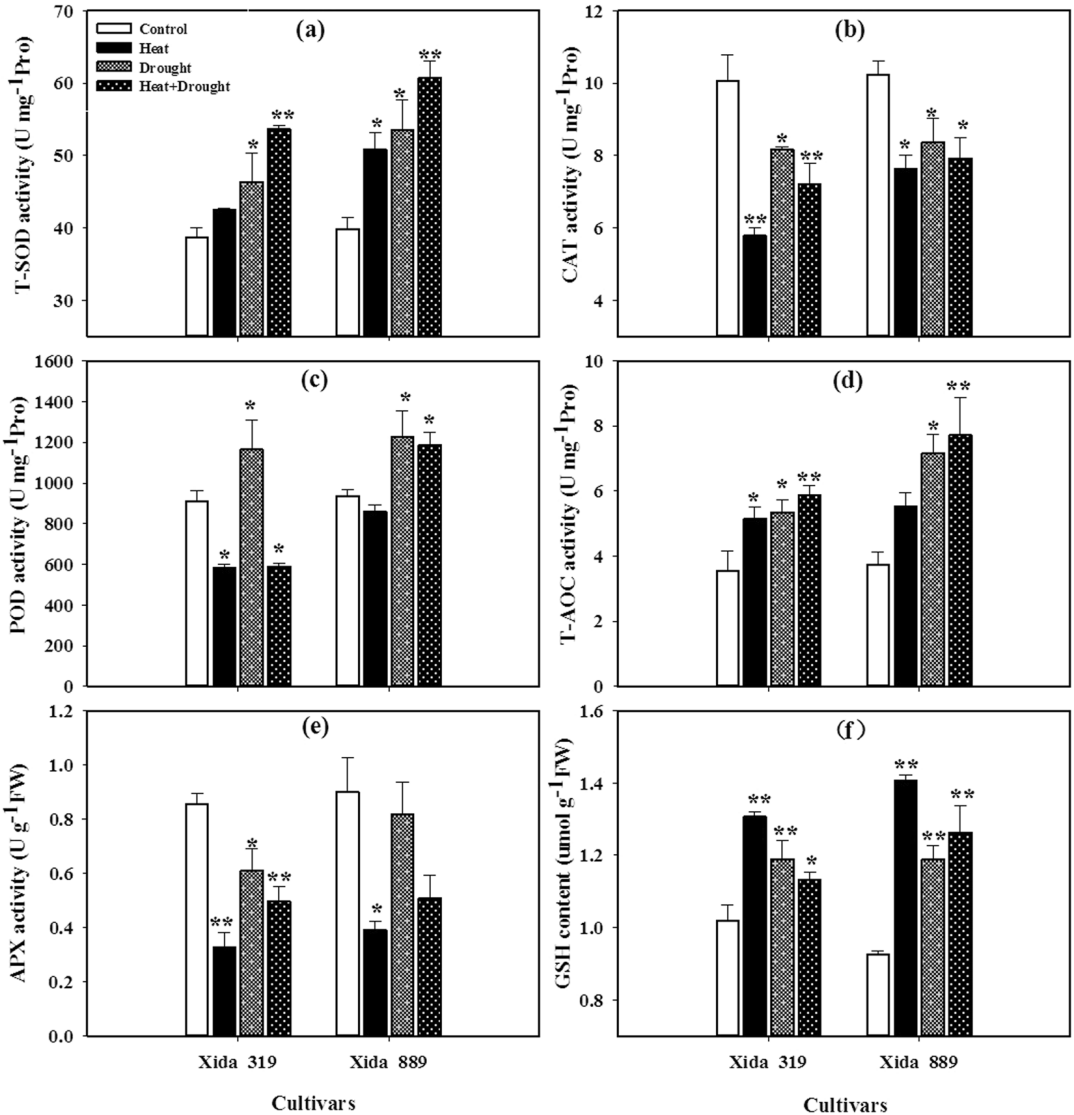
Influence of heat, drought, and heat + drought stresses on the activities/levels of **(a)** total superoxide dismutase (T-SOD), **(b)** catalase (CAT), **(c)** peroxidase (POD**), (d)** total antioxidant capacity(T-AOC), **(e)** ascorbate peroxidase (APX) and **(f)** reduced glutathione (GSH) in two maize hybrids. Capped bars above means represent ± SE of three replicates. Asterisks above columns means denote the significant differences compared with control treatment for a single maize hybrid. *P ≤ 0.05; **P ≤ 0.01.

In both maize hybrids, T-SOD, GSH and total antioxidant capacity (T-AOC) were significantly increased under stress conditions as compared with control (Fig. 4a,d,f). The enhancement of T-SOD and T-AOC was more by combined drought + heat stress as compared with their individual effects. The activities of T-SOD and T-AOC in Xida 889 were significantly higher than Xida 319 under different stress conditions. Moreover, the levels of GSH varied between two maize hybrids. As compared with control, GSH contents were increased by 28, 17 and 11% in Xida 319 and 52, 28 and 36% in Xida 889 under heat, drought and drought + heat stress conditions, respectively (Fig. 4f). However, the activities of CAT and APX in Xida 319 and in Xida 889 were significantly decreased under stress conditions, compared to control condition (Fig. 4b,e). The severe decline of CAT, POD and APX occurred under heat stress, followed by drought and drought + heat combine stress. The responses of POD to heat stress and drought stress were variable. The maximum activities of POD were recorded at drought stress in two maize hybrids (Fig. 4c).

### Osmolytes accumulation

**Figure 5:**
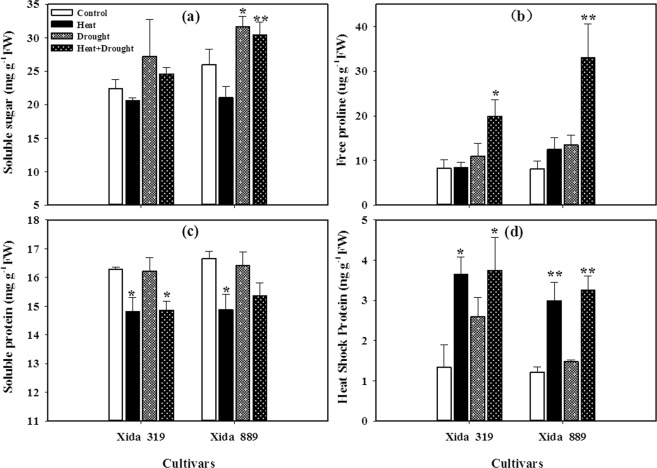
Influence of heat, drought, and heat + drought stresses on the accumulations of **(a)** soluble sugar, **(b)** free proline, **(c)** soluble protein and **(d)** heat shock protein in two maize hybrids. Capped bars above means represent ± SE of three replicates. Asterisks above columns means denote the significant differences compared with control treatment for a single maize hybrid. *P ≤ 0.05; **P ≤ 0.01.

Drought and heat stresses regulated the accumulation of soluble sugars, free proline, and heat shock protein in hybrid maize hybrids (Fig. 5). Exposure of drought, and drought + heat enhanced the soluble sugar, free proline and heat shock protein in both maize hybrids as compared with control. In contrast, the heat stress decreased the contents of soluble sugar as compared with control, but these effects were statistically non-significant. However, the concentration of soluble protein was decreased under drought, heat and drought + heat stress conditions, as compared with control. The response of heat shock protein to heat stress was greater than that to drought stress, and the maximum accumulation of heat shock protein was observed under combined drought + heat stress. Compared with Xida 319, Xida 889 showed slightly higher accumulation osmolytes, which indicated that Xida 889 may perform better under osmotic stress (Fig. 5a–d).

### Nutrient uptake

Compared with control treatment, heat stress did not significantly affect nitrogen (N) concentrations in root, leaf, and stem of both maize hybrids, while drought stress alone was found to significantly decrease the leaf N concentrations in both hybrids (Table 3). The N concentrations in root and stem of both maize hybrids under drought stress were statistically similar with those in control treatment. Under these stress treatments, N concentrations in different plant parts of maize hybrids were in the order of root N > leaf N > stem N. Exposure of drought or heat stress individually did not significantly alter the phosphorus (P) and potassium (K) concentrations in the roots and leaves of both maize hybrids with respect to control. However, drought stress significantly reduced the stem P and stem K concentrations in Xida 889 (Table 3). The combined effect of drought + heat stress was more detrimental regarding the uptake of N, P, and K for both maize hybrids. Compared with control, root N, root K, stem P, stem K, and leaf K concentrations were significantly reduced in both maize hybrids under combined drought + heat stress (Table 3). Among different plant parts, the P and K concentrations in root were lower than those in stem and leaf for both maize cultivars. The concentrations of N, P and K in the root, stem and leaf of Xida 319 were lower than Xida 889 which indicates that Xida 319 accumulated less nutrients than Xida 889, under stress conditions (Table 3).

**Table 3:** Effect of drought and heat stresses on nutrient concentrations in root, stem, and leaf of maize hybrids.

Maize cultivars	Treatments	Root			Stem			Leaf		
		N (mg/g)	P (mg/g)	K (mg/g)	N (mg/g)	P (mg/g)	K (mg/g)	N (mg/g)	P (mg/g)	K (mg/g)
Xida 319	Ck	19.52 ± 0.81	4.73 ± 0.34	10.14 ± 0.51	13.42 ± 1.42	6.29 ± 0.17	13.74 ± 0.25	17.66 ± 0.92	5.83 ± 0.50	11.60 ± 0.81
	H	19.13 ± 0.27	4.46 ± 0.39	9.26 ± 0.48	12.76 ± 1.65	5.69 ± 0.41	13.26 ± 0.16	17.99 ± 0.86	6.28 ± 0.75	11.74 ± 0.99
	D	18.74 ± 0.21	3.97 ± 0.14	8.88 ± 0.38	10.73 ± 1.37	5.44 ± 0.25	11.20 ± 1.40	13.22 ± 0.15*	4.96 ± 0.18	9.20 ± 1.29
	D + H	15.17 ± 1.07**	3.55 ± 0.71	6.67 ± 0.33**	10.52 ± 0.96	4.92 ± 0.13**	8.81 ± 0.84**	14.47 ± 2.30	5.50 ± 1.34	7.81 ± 1.03*
	Means	18.14	4.18	8.74	11.86	5.58	11.75	15.83	5.64	10.09
Xida 889	Ck	21.62 ± 1.84	4.86 ± 0.21	10.20 ± 0.79	13.50 ± 1.34	7.16 ± 0.27	13.85 ± 0.30	17.85 ± 0.15	5.85 ± 0.22	11.24 ± 0.44
	H	20.10 ± 0.54	4.64 ± 0.26	9.81 ± 0.46	12.74 ± 1.03	5.71 ± 0.28**	13.24 ± 0.56	18.82 ± 0.68	5.98 ± 0.23	11.85 ± 0.87
	D	18.97 ± 0.62	3.92 ± 0.45	8.94 ± 0.42	12.53 ± 1.31	5.11 ± 0.35**	12.60 ± 0.38*	14.47 ± 1.18**	4.98 ± 0.42	10.60 ± 0.62
	D + H	16.65 ± 0.78*	3.63 ± 0.45	7.07 ± 0.58**	10.71 ± 0.52	4.93 ± 0.28**	10.81 ± 0.97*	16.41 ± 0.54	5.57 ± 0.26	8.81 ± 0.82*
	Means	19.33	4.26	8.98	12.37	5.73	12.62	16.89	5.59	10.62

Values are means of three replicates ±SE. For Duncan’s results, means with asterisks are considered as significant different compared with control. *P ≤ 0.05; **P ≤ 0.01.

Ck, control; H, heat stress; D, drought stress and H + D, heat + drought stress.

## Discussion

Among abiotic stresses, drought and heat stresses alone or combination are the major limiting factors for the growth and productivity of many field crops. Although drought and heat stresses may disturb the overall growth and development of crop plants, yet the reproductive growth of plants is more sensitive under these stress factors^7,32,33^. In the present experiment, drought and heat stress conditions reduced the plant height, leaf area and biomass accumulation that ultimately reduced the yield and related attributes of two maize hybrids, nevertheless, the negative effects of drought + heat stresses were more severe for both maize hybrids then their individual effects (Tables 1 and 2). These stress treatments had more serious effects on Xida 319 compared with Xida 889. Previously, reduced maize growth and severe yield losses were found when drought stress was imposed at pre-tasseling^34^ and high temperature near anthesis stage^35^. Furthermore, significant reductions in growth, biomass accumulation and yield of maize were observed under heat stress imposed at different reproductive stages^35–37^. In the past, several studies have reported the detrimental effects of a combined drought and heat stresses on the growth and yields of different cereal crops^12,14,37–41^, however, the extent of damage under these stresses varies with the severity of stress and crop growth stage^7^.

In the present study, Chl a, Chl b and total Chl contents were severely affected in maize by individual and concurrent heat and drought stresses (Fig. 1a–c). However, the impacts of heat and drought + heat stress on the Chl a, Chl b and total Chl contents were severe compared with drought stress. Chlorophyll is the main pigment for photosynthesis of plants, which is one of the physiological processes that are most sensitive to high temperature^42^. High-temperature stress promotes the degradation of chlorophyll^43^, thus reducing the acceptance of light quanta, avoiding excessive free radicals and causing damage to plants^44^. Drought stress could also reduce the leaf chlorophyll contents, which on the other hand may hamper the photosynthetic efficiency and plant growth^45–47^. Drought stress decreased the RWC more than heat stress and the significantly reduction were observed under combined drought and heat stresses (Fig. 1d), which is consistent with the results by Wang *et al*.^48^ who reported that heat and drought stresses decreased the RWC in wheat plants.

Water relations in plants are influenced by several factors including Tr, leaf water potential, leaf and canopy temperatures, and Gs, while, water losses under heat stress remain high during day time because of increased Tr, which ultimately impair certain key physiological processes in plants^7^. In the present study, responses of Pn, Tr, Gs, and Ci in both maize hybrids to heat, drought and the combination of these two stresses were different. The Pn, Tr, and Gs of maize leaves decreased greatly but Ci was increased under combined drought + heat stress than individual stresses (Fig. 2). On the other hand, under heat stress, Gs and Tr of maize leaves increased slightly but Ci was decreased (Fig. 2). Under drought conditions, closure of stomata decreases the availability of CO_2_ and thus limits the Pn in plants^47,49^. By contrast, heat stress hinders Pn mainly because of nonstomatal limitations, such as alterations in electron transport capacity and activity^50,51^. Therefore, in the present study, heat induced stomatal opening increased the Tr, but resulted in a decreased Pn (Fig. 2a–c), being consistent with other reports^52–54^. Moreover, Gs is not always linked with photosynthetic capacity of plants, particularly under severe and combined stresses^49,55,56^.

Drought and heat stress could lead to over production of ROS which deteriorate photosynthetic components in plant. Enhanced O^2−^, H_2_O_2_, OH^−^ and MDA contents in both maize hybrids were observed under drought, heat and drought + heat stresses which indicate the occurrence of oxidative stress. The level of ROS and MDA was higher under drought + heat stress than their individual effects in maize seedlings (Fig. 4). Concentration of MDA contents is an indicator of extent of lipid peroxidation under stress conditions^24,43,57^. Under unfavorable environmental conditions, higher lipid peroxidation rates occur in plants because of over-production of ROS^50,58^. In general, when the rate of ROS production exceeds the anti-oxidant enzyme activities then caused damage to essential cellular components^4,59^. Plants use the complex antioxidant defense systems to overcome the uncontrolled production of ROS and protect the plants from oxidative damage^50,57,58,60^. However, the balance between ROS generation and antioxidant enzyme activities is vital to all plant species under stressful conditions^61^. Under the stress condition, SOD can disproportionate the O^2−^ produced by plants to H_2_O_2_ and O_2_ to reduce the injury of plant^62^. At individual stress level, the O_2_^−^ contents were higher under heat stress, while H_2_O_2_ contents were higher under drought stress (Fig. 3). The higher levels of O_2_^−^ in both maize hybrids under heat stress (Fig. 3) were concomitant with lower activities of T-SOD (Fig. 4). Both CAT and POD have the ability to scavenging H_2_O_2_, and the activity of CAT enzyme is high, but the affinity for H_2_O_2_ is weak. POD has a high affinity for H_2_O_2_, but it can participate in the degradation of chlorophyll and cause membrane lipid peroxidation^63^. In the present study, the contents of T-SOD, T-AOC and GSH were increased (Fig. 5a,d,f), POD was variable (Fig. 5c) and CAT and APX were significantly decreased (Fig. 5b,e), in both maize hybrids under heat, drought and heat + drought stress conditions which were consistent with overproduction of ROS and poor growth performance of these hybrids.

Proline, soluble protein and soluble sugar are the main osmotic regulators in plants. Under stress conditions, proline can be used as a solute to regulate changes in the cell’s water environment, reducing the effects of high temperature and drought stress on plant water deficit. Proline accumulated under stress conditions can also serve as a repository for energy and ammonia sources, directly participating in plant metabolism after stress relief ^64,65^. In current study, concentrations of soluble sugar and proline were considerably higher in both maize hybrids under stress conditions as compared with control, however, soluble proteins was decreased under all stress conditions, being consistent with Wang *et al*.^48^ report. Moreover, Xida 889 accumulated more osmolytes than Xida 319 (Fig. 3a–c). In this study, response of heat shock protein to heat stress was greater than that to drought stress, and the greater heat shock protein was observed with exposure to the drought + heat stress. Heat shock proteins play vital role in abiotic stresses particularly under temperature stress^66^. Several studies regarding the effects of a combination of drought and heat stress on the heat shock protein of tobacco^11^, wheat^67^ and Arabidopsis^53^ have suggested that the effect of combined stresses on plants was different from that of heat or drought stress applied individually.

The absorption, accumulation and distribution of N, P and K in various organs of maize affect growth and productivity^68^. Changes in temperature may modify the plant-nutrient relationships; however, such effects are difficult to generalize because these effects vary with different physiological processes and plant organs^69^. In the present study, heat stress alone did not significantly affect the N, P, and K concentrations in all plant parts of both maize hybrids, only stem P concentration in Xida 889 was significantly reduced compared with control (Table 3). Previously, Tingey *et al*.^70^ reported that high temperature did not change the total plant N absorption of plants in Douglas-fir (*Pseudotsuga menziesii*), however, the concentrations of N in roots and woody tissues were decreased with respect to control. Drought stress alone was found to significantly decrease the leaf N concentrations in both hybrids and stem P and stem K concentrations in Xida 889, compared with control (Table 3). In the past, several authors have documented the drought-evoked decreases in uptake and transfer of macro nutrients (N, P, and K) in various plant species^71–74^. The detrimental effects of combined drought and heat stresses were more severe regarding nutrient absorption than the individual effects of these stresses. Compared with control, combined drought + heat stress significantly reduced root N, root K, stem P, stem K, and leaf K concentrations in both maize hybrids (Table 3), which might be attributed to reduced availability, uptake, and transfer of these nutrient elements under stressful conditions. Recently, Fahad *et al*.^7^ concluded that both drought and heat stresses, particularly in combination, negatively affect the nutrient cycling, uptake, availability, and utilization in plants by disrupting different physiological functions.

## Materials and Methods

### Growth conditions and plant material

The experiment was carried out in controlled green house at the College of Agronomy and Biotechnology, Southwest University, Chongqing, China (longitude 106°26′02″E, latitude 29°49′32″N, and altitude 220 m) during spring 2017. The seeds of two maize hybrids i.e., Xida 889 and Xida 319 were obtained from Maize Research Institute, College of Agronomy and Biotechnology, Southwest University, Chongqing, China. Seeds were planted in plastic pots (24 cm in depth, 34 cm in diameter) filled with sandy-loam soil and farmyard manure in a proportion of 7:3. The total weight of each filled pot was 11 kg. In each pot, five seeds were initially sown and after emergence, two plants were maintained in each pot. Fertilizer containing urea, KCl and P_2_O_5_ (10:6:6 respectively), was applied 5 g per pot prior to sowing.

### Stress treatments

The maize plants were maintained up to tasseling stage under normal conditions. At the tasseling stage, plants were subjected to heat, drought, and combined stress treatments for a period of 15 days. For well-watered/control (Ck), and drought (D) stress conditions, the temperature was 28 °C/20 °C (day/night); while for heat (H) and drought + heat stress (D + H) treatments, temperature was set at 38 °C/30 °C (day/night). The relative humidity was in the range of 55–70%. The drought stress was imposed at 50% field capacity (FC). The drought stress treatments were regularly checked and maintained using a moisture meter TRIME-EZ/-IT (IMKO Micromodultechnik GmbH, Germany). The experimental treatments were arranged in completely randomized design (CRD) under factorial arrangement. Each treatment was replicated three times, and there were five pots in each replicate.

### Biochemical assays

Fully expanded, healthy, and undamaged plant leaves (fourth from the top) from each replicate were sampled at 15 days after imposition of stress treatments. After washing with pure distilled water, plant leaves were frozen in the liquid N_2_ and stored at −80 °C for measuring different biochemical analysis.

### Measurement of growth and yield parameters

Leaf area (LA) of maize plants was assessed following the method of Montgomery *et al*. (1911): LA = L × W × 0.75, where L indicates leaf length, W represents the maximum leaf width, and 0.75 is the factor used for determination of leaf area in maize. Plant height was measured using a meter scale, while electronic weighing balance was used to assess the shoot fresh and dry weights. Digimatic caliper (500-197-30, Mitutoyo group, Japan) was used to measure stem diameter. For the analysis of yield parameters, six plants from each replication were randomly sampled and harvested at maturity. After sun-drying, the ears were manually shelled and yield constituents such as number of ears per plant, number of kernels rows per ear and grain yield per plant were recorded. The HI was calculated as the percent ratio of grain yield and biological yield.

### Photosynthetic components and water relations

Photosynthetic components were recorded on intact leaves from the forth branch from the top, at 15 days after imposition of stress treatments. The Chlorophyll concentrations (Chl a, Chl b and total Chl) were determined according to Peng and Liu^75^. Extraction of 250 mg leaf without vein (leaf blade) was done with 10 ml ethanol-acetone (vol. 1:2), and the extract was transferred to 15 ml tube. The tubes were placed in dark to avoid light for 24 hours. The absorbance was measured at 645 nm, 663 nm, and 652 nm. The chlorophyll content was computed by the following formulae:

$$\begin{array}{rcl}{\rm{Chlorophyll}}\,{\rm{a}}\,{\rm{content}}\,({\rm{mg}}/{\rm{g}}\,{\rm{tissue}}) & = & (12.7{\rm{D}}663-2.69{\rm{D}}645)\times {\rm{V}}/(1000\times {\rm{W}})\\ {\rm{Chlorophyll}}\,{\rm{b}}\,{\rm{content}}\,({\rm{mg}}/{\rm{g}}\,{\rm{tissue}}) & = & (22.7{\rm{D}}645-4.68{\rm{D}}663)\times {\rm{V}}/(1000\times {\rm{W}})\\ {\rm{Total}}\,{\rm{chlorophyll}}\,({\rm{mg}}/{\rm{g}}\,{\rm{tissue}}) & = & {\rm{D}}652\times {\rm{V}}/(34.5\times {\rm{W}})/{\rm{Chl}}\,{\rm{a}}+{\rm{Chl}}\,{\rm{b}}\end{array}$$

where, D663, D645 and D652 respectively are the corresponding wavelengths of the light density value, V is extracting liquid volume and W is leaf fresh weight.

Relative water content of maize leaves was measured following the method of Barrs and Weatherley^76^. The fresh leaves from the uppermost branch were sampled from plants and were cut into small segments (1.5 cm length), weighed (FW: fresh weight), floated on distilled water for 3 h under low light, and weighed again for turgid weight (TW), after surface moisture was removed. Afterward, the leaf samples were oven dried at 80 °C for 24 h and dry weight (DW) was recorded. The RWC was calculated as follows:

$${\rm{RWC}}( \% )=({\rm{FW}}-{\rm{DW}})/({\rm{TW}}-{\rm{DW}})\times 100$$

The photosynthetic gas exchange attributes including net photosynthesis rate (Pn), stomatal conductance (Gs), transpiration rate (Tr), and intercellular CO_2_ concentration (Ci) were recorded using a portable infrared gas exchange analyzer (Li-6400, Li-Cor, Lincoln, Nebraska, USA).

### Lipid peroxidation rate and ROS accumulation

Lipid peroxidation in maize leaves was determined as MDA content; measured by thiobarbituric (TBA) method using ‘MDA Kit (A003-3)’ obtained from Suzhou Comin Biotechnology Co., Ltd., China. Lipid hydroperoxide decomposition products can condense with thibabituric acid (TBA) to produce red compounds. The absorbance for MDA was measured at 532 nm and represented as nmol/g fresh weight.

The contents of H_2_O_2_ and OH^−^ in the leaves of maize were measured using the commercial ‘H_2_O_2_ Kit (H_2_O_2_-Y)’ and ‘OH^−^ Kit (QZQ-G)’, respectively, obtained from Suzhou Comin Biotechnology Co., Ltd., China. H_2_O_2_ content was measured at 415 nm and expressed as μmol/g fresh weight. The ability to remove hydroxyl radicals measured at 536 nm and expressed as percent content. The contents of O_2_^−^ in the leaves of maize were noted using the commercial ‘O_2_^−^ kit (A052)’, obtained from Nanjing Jiancheng Bioengineering Institute, China. The O_2_^−^ content was demonstrated as unit’s g^−1^ fresh weight and one unit was equivalent to superoxide anion radical inhibition by 1 mg of Vc for 40 minutes at 37 °C reaction^24^.

### Estimation of enzymatic and non-enzymatic antioxidants

The activities of antioxidant enzymes were recorded using commercial kits in accordance with the instructions of manufacturer. The kits for total superoxide dismutase (T-SOD, A001), catalase (CAT, A007) and ascorbate peroxidase (APX, A123) were procured from Nanjing Jiancheng Bioengineering Institute, China. The absorbance readings of T-SOD, CAT and APX were detected at 550 nm, 405 nm and 290 nm, respectively. The T-SOD and CAT activities in maize leaves were articulated as U/mg protein, while APX activity was demonstrated as U/g protein. One unit (U) of T-SOD was the amount of enzyme required for 1 mg tissue proteins in 1 ml of a reaction mixture to raise SOD inhibition rates to 50% at 550 nm. One unit of CAT activity was estimated as the amount of enzyme that decomposes 1μmol H_2_O_2_ at 405 nm sec^−1^ in 1 mg tissue proteins. One unit of APX was defined as the amount of enzyme required for catalyzing 1μmol ASA at 290 nm min^−1^ of 1 mg tissue proteins in 1 ml of a reaction mixture.

The POD activity, GSH content and T-AOC in the leaves of maize were determined using the commercial kit ‘POD (POD-Y)’, ‘GSH (GSH-W)’ and ‘T-AOC (TAOC-G)’, respectively, obtained from Suzhou Comin Biotechnology Co., Ltd., China. One unit of POD activity was defined as the absorbance change of 0.005 at 470 nm min^−1^ for 1 mg tissue proteins in 1 ml of a reaction mixture. The absorbance for GSH was measured at 412 nm and expressed as μmol/g fresh weight. The absorbance for T-AOC was measured at 593 nm and expressed as U/mg protein. One unit of T-AOC was expressed as the amount that enhanced the absorbance by 0.01 at the 37 °C.

### Osmolyte accumulation profiles

Free proline contents were assessed by following the acid ninhydrin method^77^. Fresh leaf material (0.5 g) was extracted using 5 ml of 3% sulphosalicylic acid for 10 min with shaking at 100 °C. The 2 ml of filtered aqueous extract was mixed with glacial acetic acid (2 ml) and acid ninhydrin reagent (2 ml), and heated (100 °C) for 30 min. The reaction mixture after cooling was segregated against toluene (4 ml) and the absorbance of the organic phase was recorded at 520 nm. The resulting values were related with a standard curve plotted using known amounts of proline (Sigma, St Louis, MO, USA). Total soluble sugar was estimated by anthracene ketone method as described by Zong and Wang^78^. The fresh leaf sample (0.2 g) was homogenized with 25 ml distilled water and centrifuged (4000 rpm) for 20 minutes. Anthracene (0.1 g) was dissolved in 100 ml diluted sulfuric acid to prepare anthracene sulfuric acid reagent. One ml extract and 5 ml anthracene sulfuric acid reagent were taken in a tube, shaken and put in boiling bath for 10 minutes. After 2 h stability, the sample was transferred in cuvette and the absorbance was read at 620 nm. The total protein content in the leaves of maize seedling was determined by Coomassie brilliant blue method using ‘Protein Quantification Kit (A045-2)’ obtained from Suzhou Comin Biotechnology Co., Ltd., China. The absorbance for protein was measured at 595 nm and unit of protein was quantified as mg/ml sample solution. The level of heat shock protein (HSP) in the leaves of maize was determined using the kit ‘HSP (YX-081916P)’ obtained from Sino Best Bio, Shanghai. The absorbance for HSP was measured at OD 450 nm and expressed as pg/ml sample solution.

### Determination of nutrient uptake

Roots, stems and leaves were thoroughly washed with distilled water and were oven-dried at 105 °C for 1 hour and later at 80 °C until constant weight. Dry samples of 300 mg were digested with 8 ml sulfuric acid (H_2_SO_4_) in a sealed chamber^79^. Total N was determined using the Kjeldahl method^80^. The K level was analyzed using an Elemental Analyzer, while P concentration was determined by vanadate molybdate method using a UV/visible spectrophotometer as suggested by Chapman and Pratt^81^.

### Statistical analysis

The collected data were statistically analyzed following analysis of variance (ANOVA) technique using SPSS 22.0 software (SPSS, Chicago, IL, USA) whereas the differences between control and stress treatments were separated according to Duncan’s multiple range test at a 1% and 5% level of significance. SigmaPlot 13.0 software (Systat Software Inc., San Jose, CA, USA) was used for the graphical presentation of data.

## Conclusions

In summary, exposure of drought, heat and drought + heat stress at tasseling stage brought severe negative effects on growth and the yield attributes of the maize hybrids. Concurrent effects of drought + heat stress on photosynthetic components, osmolyte accumulation, enzymatic and non-enzymatic antioxidants and nutrients uptake of both maize hybrids were more severe than their individual effects. Under drought and heat stress conditions, higher ROS accumulation and rate of lipid peroxidation inhibited the photosynthetic efficiency and restricted the growth of maize. The levels of ROS scavengers were variable in both maize hybrids. Overall, the morpho-physiological growth, and yield performance of Xida 889 cultivar was better than the Xida 319 cultivar. The greater tolerance of Xida 889 to heat and drought stresses was attributed to strong antioxidant defense system, higher osmolyte accumulation, and maintenance of photosynthetic pigments and nutrient balance compared with Xida 319.
